# Sporulation Abilities and Heat Resistance of *Clostridium perfringens* Strains Isolated from French Food Borne Outbreaks

**DOI:** 10.3390/foods14213735

**Published:** 2025-10-31

**Authors:** Olivier Firmesse, Véronique Maladen, William Bourelle, Michel Federighi, Christina Tran, Narjes Mtimet

**Affiliations:** 1 Staphylococcus, Bacillus & Clostridium Unit, Laboratory for Food Safety, ANSES, 94700 Maisons-Alfort, France; olivier.firmesse@anses.fr (O.F.); william.bourelle@inrae.fr (W.B.); christina.tran@anses.fr (C.T.); 2 Ecole Nationale Vétérinaire d’Alfort, 94700 Maisons-Alfort, France; 3 Salmonella and Listeria monocytogenes Unit, Laboratory for Food Safety, ANSES, 94700 Maisons-Alfort, France

**Keywords:** *Clostridium perfringens*, sporulation, heat resistance, CPE, chromosomal *cpe* (c-*cpe*), plasmidic *cpe* (p-*cpe*)

## Abstract

*Clostridium perfringens* is responsible for various diseases. Foodborne outbreaks (FBOs) result from the in situ production of *C. perfringens* enterotoxin (CPE) by type F strains during sporulation. The *cpe* gene can be plasmidic (p-*cpe*) or chromosomal (c-*cpe*). Strains (c-*cpe*) exhibit greater heat resistance and are frequently associated with FBO. Strains *cpe*-negative are considered heat-sensitive. This study investigates the sporulation abilities and heat resistance of eight *C. perfringens* strains isolated from French foodborne outbreaks. Whole-genome sequencing classified the strains into two clades: the “chromosomal *cpe* clade,”, mainly composed of *cpe*-positive strains with c-*cpe* and some *cpe*-negative strains, and the “plasmidic *cpe* clade,”, primarily containing *cpe*-negative strains and a few with plasmid-borne *cpe*. Sporulation assays and thermal inactivation kinetics were performed on FBO strains to evaluate the influence of genetic variability on sporulation abilities and heat resistance. Experimental analyses revealed that strains within the “chromosomal *cpe* clade” exhibited the highest sporulation abilities, regardless of *cpe* presence, while those in the “plasmidic *cpe* clade” had low sporulation ability. Moreover, heat-resistant spores were produced exclusively by strains of the “chromosomal *cpe* clade,” with c-*cpe* strains exhibiting the highest heat resistance (δ_95 °C_ ≈ 49 min), followed by *cpe*-negative strains (δ_95 °C_ ≈ 9.5 min). p-*cpe* strains exhibited a heat-sensitive phenotype, with δ_85 °C_ values of 12 min. A key finding of this study is the identification of a group with intermediate heat resistance, distinct from the highly heat-resistant (c-*cpe*) and heat-sensitive (p-*cpe*) strains. This intermediate heat-resistance phenotype, observed in *cpe*-negative strains within the “chromosomal *cpe* clade,” offers a new perspective on *C. perfringens* stress adaptation, suggesting their potential for persistence in food. Their heat resistance, along with the potential for *cpe* gene transfer, could make these strains a relevant hazard for cooked, cooled, and re-heated meat products.

## 1. Introduction

*Clostridium perfringens* is a Gram-positive, spore-forming anaerobic bacterium found in various environments and is responsible for a variety of human and animal diseases. The ability of *C. perfringens* to cause disease in various hosts is primarily attributed to its arsenal of virulence factors, including toxins and enzymes [1,2]. The production patterns of six toxins (α-toxin, β-toxin, ε-toxin, ι-toxin, CPE, and NetB) among different strains form the basis for a recently revised classification scheme [3]. This new classification scheme categorizes *C. perfringens* isolates into one of seven toxinotypes from A to G (Table 1), which improves epidemiological and diagnostic value in infections in both humans and animals [3]. The toxin-based typing method for *C. perfringens* has shown that different toxinotype strains have distinct host preferences and are linked to specific diseases (Table 1) [2,3]. *C. perfringens* type F produces the α-toxin and, upon sporulation, also produces the *C. perfringens* enterotoxin (CPE), which is responsible for food poisoning (FP).

CPE is considered the main toxin responsible for symptoms in foodborne illnesses caused by *C. perfringens* [4]. This enterotoxin is produced by all type F strains and by some type C, D, and E strains [2,3].

**Table 1:** *C. perfringens* toxinotype classification scheme and disease association.

**Toxinotype**	**α-Toxin, CPA** **(*plc* or *cpa*)**	**β-Toxin, CPB** **(*cpb*)**	**ε-Toxin, ETX** **(*etx*)**	**ι-Toxin, ITX** **(*iap* and *ibp*)**	**CPE** **(*cpe*)**	**NetB** **(*netB*)**	**Main Diseases and Affected Species**
A	+	−	−	−	−	−	Gas gangrene in humans and several animals; possible involvement in enterotoxemia and GI disease in ruminants, horses, and pigs; hemorrhagic gastroenteritis in dogs and horses
B	+	+	+	−	−	−	Lamb dysentery
C	+	+	−	−	+/−	−	Hemorrhagic and necrotizing enteritis in several neonatal animals; struck; enteritis necroticans (pig-bel, Darmbrand) in humans
D	+	−	+	−	+/−	−	Enterotoxemia in sheep, goats, and cattle; enterocolitis in goats
E ^(^*^)^	+	−	−	+	+/−	−	Possible involvement in gastroenteritis of cattle and rabbits
F	+	−	−	−	+	−	Human food poisoning, antibiotic-associated diarrhea and sporadic diarrhea
G	+	−	−	−	−	+	Necrotic enteritis in poultry

^(^*^)^ Type E strains either carry a functional or a silent *cpe* gene [5]; (+) toxin produced; (−) toxin not produced.

Food poisoning can occur when food contaminated with a high concentration of vegetative cells (i.e., >10^6^ to 10^7^ vegetative cells/gram of food) from a type F strain of *C. perfringens* is ingested [6]. Many of these bacteria are destroyed by gastric acids but some survive and reach the intestines. After a growth phase, *C. perfringens* undergoes sporulation and synthesizes CPE. The exact triggers for in vivo sporulation are not fully understood, but may involve exposure to stomach acidity, bile salts, or phosphate in the intestines [6,7].

Approximately 5% of global *C. perfringens* isolates carry the *cpe* gene and produce CPE, making the isolation of *cpe*-positive strains relatively uncommon [4,8,9,10,11]. The *cpe* gene can be located either on plasmids or on a transposable element integrated into the chromosome, and both plasmid-mediated (p-*cpe*) and chromosomal (c-*cpe*) strains can cause foodborne outbreaks (FBOs) [9,10]. Approximately 70% of type F human food poisoning isolates carry their *cpe* gene on the chromosome. The remaining 30% of food poisoning strains, and virtually all non-foodborne human gastrointestinal (GI) diseases *cpe*-positive strains, carry their *cpe* gene on plasmids [4,12]. A recent study [1] conducted on a collection of 141 strains of *C. perfringens* detected in 42 FBOs in the Paris region have shown that 79 strains were assigned to *C. perfringens* type F (56%), 61 strains were assigned to *C. perfringens* type A (43%), and one strain was assigned to *C. perfringens* type E (1%). Genomic analysis performed on 58 strains revealed that *C. perfringens* strains carrying the *cpe* gene on the chromosome (24/58) are more abundant compared to strains with plasmid-mediated *cpe* (3/58), confirming the prominent implication of chromosomal *cpe* (c-*cpe*) strains in human food poisoning [1,12]. Many studies suggest that the specific association between chromosomal *cpe* isolates and *C. perfringens* type F food poisoning is partly due to the significantly greater heat resistance of chromosomal *cpe* isolates compared to plasmid *cpe* isolates [8,9]. This enhanced heat resistance allows chromosomal *cpe* isolates to survive better in cooked meat products, which are the major vehicle implicated in *C. perfringens* type F outbreaks [1,13,14].

Several known mechanisms contribute to the heat resistance of spores within *Clostridia* species, particularly *C. perfringens*, which share similarities with the spores of *Bacillus subtilis* [15,16,17]. The main factor responsible for spore heat resistance is the low water content of the protoplast or the spore core [18,19,20]. In addition to the water content of the protoplast, other factors may contribute to spore resistance: (i) The accumulation of dipicolinic acid (DPA) and its chelated bivalent cations (Ca^2+^) in the spore core helps reduce its water content during sporulation and consequently increases resistance to wet heat [21,22,23]. (ii) The nature of the mineral cation associated with DPA. In fact, spores formed in the presence of Ca^2+^ exhibit greater protection against moist heat compared to those formed with other divalent cations (Mg^2+^, Mn^2+^) or monovalent cations (K^+^, Na^+^) [21,24]. (iii) The saturation of DNA with small acid-soluble spore proteins (SASPs) which protect DNA from heat [19,20]. SASPs are a class of low-molecular weight proteins that bind to spore DNA and provide protection from various environmental stresses. For *C. perfringens* food poisoning strains, a variant allele of small acid-soluble proteins *ssp4* was associated with spore’s heat resistance [12,25]. However, a recent study has reported a heat-resistant phenotype for a c-*cpe* strain without the presence of heat resistance-associated allele of Ssp4 [9].

Although the sporulation and heat resistance of *C. perfringens* have been investigated in previous studies, none have compared sporulation abilities between *cpe*-negative strains and *cpe*-positive strains (c-*cpe* and p-*cpe*). Moreover, in the reviewed literature, it seems that *cpe*-negative strains have generally been considered as heat-sensitive, with studies mainly focusing on heat resistance comparison between c-*cpe* and p-*cpe* strains [9,26,27]. Accordingly, the present study aims to examine the sporulation abilities and the heat-resistant characteristics of eight strains of *C. perfringens* detected in French FBO (*cpe*-negative and *cpe*-positive strains). A comparison was made with other studies to examine the link between sporulation, heat resistance, and the presence or absence of the *cpe* gene and its location (on the chromosome or on the plasmid).

The last sentence of the paragraph refers exclusively to *C. perfringens*.

## 2. Materials and Methods

### 2.1. Bacterial Strains

A total of eight *C. perfringens* strains isolated from foods involved in French FBOs from 2016 to 2017 were selected and used for sporulation and heat resistance assays (Table 2). Strains were classified and selected according to their affiliation with either the “chromosomal *cpe* clade” or to the “plasmidic *cpe* clade”. The “chromosomal *cpe* clade” is a clade that contains mainly *cpe*-positive strains carrying the *cpe* gene on a chromosome (c-*cpe*) and few strains in which the *cpe* gene is absent [1]. The “plasmidic *cpe* clade” is a clade that contains mainly *cpe*-negative strains and few *cpe*-positive strains with plasmid-mediated *cpe* (p-*cpe*) [1]. The phylogenetic tree was published in 2019 [1]. *C. perfringens* NCTC 8239 (ATCC 12917; CIP 104880), originally isolated from boiled salted beef, was purchased from the Pasteur Institute collection and used in this study as a control strain for sporulation and heat resistance essays. This strain carries a chromosomal *cpe* (c-*cpe*) [7,15].

### 2.2. Media Preparation for Growth and Sporulation

Tryptone–Peptone–Glucose (TPG) supplemented with sodium thioglycolate was used as liquid growth media for all strains of *C. perfringens*. Tryptone and peptone were purchased from Oxoid (Lyon, France). Glucose and sodium thioglycolate were purchased from Oxoid (France) and Merck (Lyon, France), respectively. For sporulation of *C. perfringens*, modified Tryptone–Peptone–Glucose (m-TPG) medium was used. Tryptone–Peptone–Glucose used for sporulation was supplemented with sodium thioglycolate (1.0 g L ^−1^, MW 114.10, Merck, France), MnSO_4_ (5.0 mg L−1, MW 169.02, Sigma-Aldrich, Fallavier, France) and CaCl_2_ (5.0 mg L−1, MW 110.99, Thermoscientific, Illkirch, France). The pH was adjusted to 7.5 with NaOH (1 M) in the presence of phosphate-buffered saline (KCl 2.7 mM, NaCl 140 mM, Phosphate 10 mM, PanReac AppliChem, Darmstadt, Germany). Glass jars containing sporulation media (m-TPG) were sealed and autoclaved at 121 °C for 15 min. Finally, sodium taurocholate (MW 537, Merck, France) was added after sterilization at a final concentration of 500 µM.

### 2.3. Spore Production

For all experiments, a first pre-culture was obtained from a vegetative cell stock of cryogenic beads (CRYOBEADS, bioMérieux, Marcy-l’Étoile, France). This first pre-culture was carried out by adding a cryogenic bead to 9 mL of TPG contained in a sealed jar in the presence of an anaerobic generator (GENbox anae, bioMérieux, France) at 37 °C for 24 h to reach an approximate concentration of 7.0 log_10_ CFU/mL. An anaerobe indicator (Anaer Indicator, bioMéreux, France) was introduced in sealed jars with media flasks to check anaerobiosis. A second pre-culture was carried out by inoculation of 9 mL of TPG with 100 µL of the first pre-culture, followed by anaerobic incubation at 37 °C for 6 h. The second pre-culture (300 µL) was used to inoculate 30 mL of sporulation media; then, sporulation was conducted for three days at 37 °C under anaerobic conditions. Spores were harvested by centrifugation, washed three times (3000 rpm for 15 min) in sterile ultrapure water (Millipore Simplicity Water Purification System, Merck, France) and kept at 4 °C. Sporulation was verified using a phase-contrast microscope (Olympus BX51, Olympus, Rungis, France, 10 × 100 Oil). To deactivate vegetative cells and ensure that only spores were recovered and counted, the produced spore suspension was heat-treated in a water bath at 75 °C for 15 min. Spore concentration was evaluated after the heat treatment by plating on Columbia agar + 5% sheep blood (bioMérieux, France).

### 2.4. Heat Resistance Assays

Inactivation kinetics were performed at 85 °C or 95 °C according to the capillary tube method, allowing heat treatment in isothermal conditions. Glass capillary tubes with a 200 µL capacity (Hirschman Laborgerate, Germany) were filled with 100 µL of spore suspension and then sealed at both ends with a gas burner flame. The capillary tubes were heated at 85 °C or 95 °C in a thermostated glycerol/water bath with a circulating-water pump to maximize heat exchange. Immediately after heat treatment, the glass capillary tubes were placed in melting ice. The glass tubes were washed, broken, and poured into 0.9 mL of tryptone salt broth [28,29]. The heat-treated spores were counted by plating on Columbia agar + 5% sheep blood after anaerobic incubation at 37 °C for 24 h.

### 2.5. Heat Resistance Estimation

Survival curves (log CFU/mL vs. heating time) were fitted by the model based on the Weibull distribution proposed by Mafart et al. (2002) [30]:

(1) $$\log\frac{N}{N_{0}}\;=\;-{\left(\frac{t}{\delta }\right)}^{p}$$

where *N* is the population size at time t (CFU/mL), *N***_0_** is the initial population size (CFU/mL), *δ* is the first decimal reduction time that led to a 10-fold reduction (min), and *p* is the shape parameter of the survivor curve. For *p* > 1, the curve is convex; for *p* < 1, the curve is concave; and for *p* = 1, the curve is log-linear. A single *p* value was estimated for the whole set of data. The initial population size, as well as the first decimal reduction time, was estimated for each survival curve.

### 2.6. Statistical Analysis

A non-linear fitting function (lsqcurvefit function, Optimization Toolbox, MATLAB R2024b, The MathWorks, Natick, MA, USA) was used to estimate *δ*-values. Goodness of fit and the performance of the inactivation model was evaluated by calculating the following indicators: the coefficient of determination (*R*^2^), the mean squared error (*MSE*), the root mean square error (*RMSE*) and the corrected Akaike Information Criterion (*AICc*). The closer *R*^2^ is to 1, the better the prediction. For *MSE* and *RMSE,* a value close to zero indicates a smaller random error component. The corrected Akaike Information Criterion (*AICc*) was calculated to evaluate the fitting performance of the model used for heat resistance estimation. A lower *AICc* reveals an appropriate model for prediction [31]. A multiple comparison procedure (anova1 and multcompare function, Statistical Toolbox, Matlab R2024b, The MathWorks, Natick, MA, USA) was implemented to assess the impact of strain variability on spore production ability and heat resistance abilities.

## 3. Results

### 3.1. Sporulation Abilities of C. perfringens Strains

**Figure 1:**
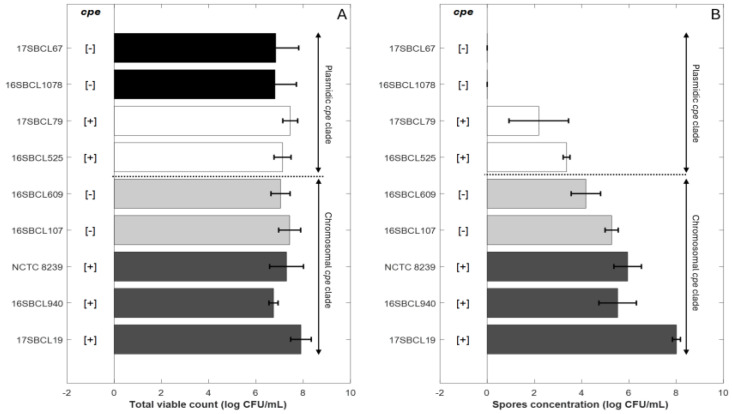
Sporulation abilities of *Clostridium perfringens* strains. Control strains NCTC 8239 (ATCC 12917/CIP 104880) and eight FBO strains were included in the study. The “chromosomal *cpe* clade” is a clade that contains mainly *cpe*-positive strains carrying the *cpe* gene on a chromosome (c-*cpe*) and few strains in which the *cpe* gene is absent [1]. The “plasmidic *cpe* clade” is a clade that contains mainly *cpe*-negative strains and few *cpe*-positive strains with plasmid-mediated *cpe* (p-*cpe*) [1]. (**A**) represents the total viable count obtained after three days of sporulation without heat treatment for the different strains. (**B**) represents the spore concentration for each strain following heat treatment at 75 °C for 15 min. Dark grey bars (c-*cpe* strains), light grey bars (*cpe*-negative strains belonging to chromosomal *cpe* clade), white bars (p-*cpe* strains), black bars (*cpe*-negative strains belonging to plasmidic *cpe* clade). [+]: presence of *cpe* gene. [−]: absence of *cpe* gene.The plot was generated in MATLAB R2024b.

The spore quantities produced by the control strains *C. perfringens* NCTC 8239 and the FBO strains were estimated. The final spore concentration obtained for each strain following heat treatment at 75 °C for 15 min (Figure 1B) was compared to the total viable count (Figure 1A) obtained after three days of sporulation without heat treatment.

The strains were classified based on their belonging to the “chromosomal *cpe* clade” or to the “plasmidic *cpe* clade” [1]. The details of the classification are provided in the Section 2.

For strains belonging to the “chromosomal *cpe* clade”, final mean spore concentrations were about 6.24 (±1.11) log_10_ CFU/mL for chromosomal *cpe*-positive strains (17SBCL19, 16SBCL940, NCTC 8239) and about 4.72 (±0.73) log_10_ CFU/mL for the *cpe*-negative strains (16SBCL107, 16SBCL609) (Figure 1.B). For strains belonging to the “plasmidic *cpe* clade”, final mean spore concentrations were about 2.77 (±1.02) log_10_ CFU/mL for plasmid-mediated *cpe*-positive strains (16SBCL525, 17SBCL79). No spores were detected in our experimental conditions for *cpe*-negative strains belonging to the “plasmidic *cpe* clade” (16SBCL1078, 17SBCL67).

The results show that the greatest spore production was generally observed for strains belonging to “chromosomal *cpe* clade” whether or not they carried the *cpe* gene. In comparison, strains belonging to “plasmidic *cpe* clade” exhibited a low ability to produce spores under our experimental conditions. A significant difference in spore production was observed between *cpe*-positive strains carrying the *cpe* gene on a chromosome (c-*cpe*) compared to (p-*cpe*) strains (*p* value < 0.05). Furthermore, within the “chromosomal cpe clade”, *cpe*-negative strains did not show a significant difference in spore production compared to *cpe*-positive strains, except for the strain 17SBSC19, which produced the highest spore concentration (7.9 ± 0.43 log_10_ CFU/mL).

### 3.2. Heat Resistance Characterization of C. perfringens Strains

To investigate the effect of strain genetic variability on heat resistance properties, thermal inactivation kinetics were performed at 85 °C or 95 °C on spore suspensions produced as described previously. Triplicates were performed for each strain from three independent spore batches. Typical survivals curves were obtained and then fitted to the Weibull model. The spore heat resistance for each strain was estimated using the first decimal reduction time (*δ*) (Table 3). The goodness of fit was evaluated by calculating the *RMSE*, *AICc,* and other statistical indicators (Table 3). The results show that the model provided a good fit to the data (34 survival curves) and accurately describes the thermal inactivation kinetics of *C. perfringens* spores. The thermal inactivation curves exhibited a concave shape, with a *p*-value estimated at 0.68 (Table 3).

**Figure 2:**
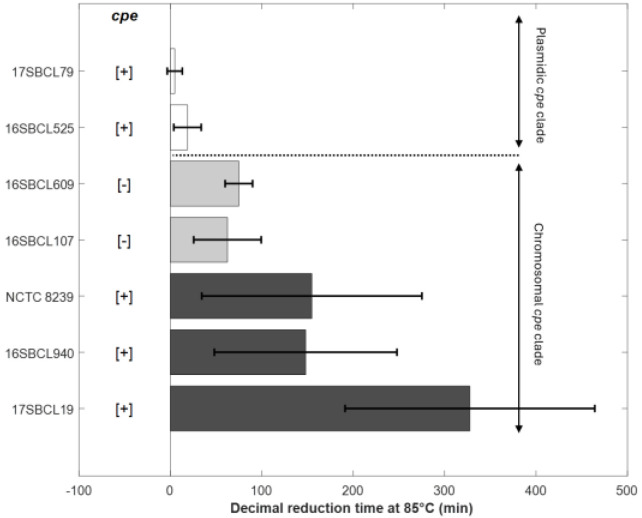
Decimal reduction time (min) obtained at 85 °C for the control strains NCTC 8239 (ATCC 12917/CIP 104880) and six FBO strains. The error bars indicate standard deviation. The “chromosomal *cpe* clade” is a clade that contains mainly *cpe*-positive strains carrying the *cpe* gene on a chromosome (c-*cpe*) and few strains in which the *cpe* gene is absent [1]. The “plasmidic *cpe* clade” is a clade that contains mainly *cpe*-negative strains and few *cpe*-positive strains with plasmid-mediated *cpe* (p-*cpe*) [1]. Dark grey bars (c-*cpe* strains), light grey bars (*cpe*-negative strains belonging to chromosomal *cpe* clade), white bars (p-*cpe* strains). [+]: presence of *cpe* gene. [−]: absence of *cpe* gene. The plot was generated in MATLAB R2024b.

To characterize the heat resistance phenotypes associated with the two clades established by Abdelrahim et al. (2019) [1], spores produced from the sequenced strains underwent heat resistance assays. As shown in Figure 2 and Table 3, *C. perfringens* strains carrying a chromosomal *cpe* gene (c-*cpe*) produced highly heat-resistant spores with *δ*_85 °C_ values over 100 min and an average of 210 min. However, strains with a plasmid *cpe* gene (p-*cpe*) produced heat-sensitive spores with δ_85 °C_ values ranging from 5 to 19 min (Table 3). A significant difference (*p* value < 0.05) was observed between c-*cpe* strain (17SBCL19) and two p-*cpe* strains (16SBCL525, 17SBCL79) (Figure 2).

Interestingly, *cpe*-negative strains belonging to the “chromosomal *cpe* clade” produced relatively heat-resistant spores with *δ*_85 °C_ values ranging from 62 to 75 min (Table 3). To further investigate the differences between *cpe*-positive and *cpe*-negative strains within the “chromosomal *cpe* clade”, we compared their *δ*_95 °C_ values (Table 3). Indeed, a heat treatment at this temperature is more suitable for studying the decimal reduction time of these heat-resistant strains. No significant difference was observed among the strains within the “chromosomal *cpe* clade,” regardless of the presence or absence of the *cpe* gene (*p* value < 0.05). Our results show that *cpe*-negative strains belonging to the “chromosomal *cpe* clade” form a group with an intermediate level of heat resistance, positioned between the highly heat-resistant strains (c-*cpe*) and the heat-sensitive strains (p-*cpe*).

We also acknowledge that under our experimental conditions, no spores were recovered after a heat treatment at 75 °C for 15 min intended to eliminate vegetative cells of *cpe*-negative strains from the “plasmidic *cpe* clade”. As a result, we were unable to assess their heat resistance.

## 4. Discussion

*C. perfringens* is a well-known spore-forming bacterium and a significant contributor to bacterial food poisoning, particularly in cooked meat products. However, it exhibits a notoriously poor ability to form spores in most laboratory media [32]. In our study, preliminary experiments revealed that the control strain’s sporulation yield (NCTC 8239) was significantly higher (approximately 3 log_10_ units greater) in the newly developed medium (m-TPG) compared to the commonly used Duncan–Strong medium. For this reason, this sporulation medium was adopted for subsequent experiments. However, we acknowledge at this stage that there is no evidence indicating that this medium is suitable for the sporulation of all *C. perfringens* strains. Many sporulation media have been developed to enhance and optimize the sporulation of *C. perfringens* [32,33,34]; but, to our knowledge, none of these media can be considered as the most suitable for the sporulation of all strains. All we can conclude is that sporulation initiation requires inorganic phosphate (Pi) in the environment and that Pi neutralizes the inhibitory effect of glucose and induces the expression of *spo0A* [35]. Another parameter that might regulate the formation of *C. perfringens* spores is the pH. Sporulation efficiency can be enhanced at a pH of 7.5–8 in the medium [32,34]. Finally, sodium taurocholate and thioglycolate have been reported to stimulate sporulation, although this effect varies depending on the strain and experimental conditions [33,36]. The significant variability observed in the sporulation conditions of *C. perfringens* strains remains fascinating and calls for further investigation.

In our experimental conditions, we observed that the greatest spore production was generally associated with strains belonging to “chromosomal *cpe* clade” regardless of the presence or absence of the *cpe* gene. The “chromosomal *cpe* clade” is a clade that contains mainly *cpe*-positive strains carrying the *cpe* gene on a chromosome (c-*cpe*) and few strains in which the *cpe* gene is absent [1]. The clonal genomic relationship of chromosomal *cpe* strains was reported and described recently [7,37]. The authors associated this clade to phylogroup I, which contains c-*cpe* strains, including our control strain *C. perfringens* NCTC 8239 and other strains in which the *cpe* gene is absent. Phylogroup I presents a distinctive feature, as its isolates exhibit a markedly smaller genome size compared to other phylogroups [7]. For *cpe*-negative strains belonging to the phylogroup I (“chromosomal *cpe* clade”), the possibility of *cpe* gene loss was suggested by the authors in [7,37]. In addition to their high sporulation ability, strains belonging to this clade have demonstrated the ability to produce heat-resistant spores. Some authors have identified two genes potentially associated with spore heat resistance in c-*cpe* strains: a specific allele of GrpE and the presence of CoA-disulfide reductase NaoX [9]. A variant allele of the small acid-soluble protein Ssp4 has also been linked to spore heat resistance [12,25]. However, a recent study reported a heat-resistant phenotype in a c-*cpe* strain lacking the heat-resistance-associated allele of Ssp4 [9]. Regarding the “plasmidic *cpe* clade”, it contains mainly *cpe*-negative strains and few *cpe*-positive strains with plasmid-mediated *cpe* [1]. For this clade, strains exhibited limited ability to produce spores, and when formed, these spores are generally heat-sensitive [9,27].

In fact, our results are in agreement with the literature and the findings obtained by other authors. Table 4 presents a non-exhaustive review of heat resistance value obtained for different *C. perfringens* strains by different authors. When log-linear model was used for heat resistance estimation, results were expressed as *D*-value in min. When model based on Weibul distribution was used for heat resistance estimation, results were expressed as *δ*-value in min. By comparing the results obtained for the reference strain NCTC 8239 (c-*cpe*), we found that the heat resistance in our study (*δ*_95 °C_ = 46 min) was similar to that reported by Mehdizadeh Gohari’s (2024) study (*D*_100 °C_ = 43.3 min) [27], despite differences in the sporulation medium (Table 4). Indeed, we used the m-TPG medium instead of the commonly used Duncan–Strong medium employed by Mehdizadeh Gohari, since our developed medium yielded more spores.

Our results confirm previous studies showing that strains carrying *cpe* chromosomally exhibit high heat resistance, enabling them to survive more effectively in improperly cooked or stored food, which also explains their significant involvement in FBO [9]. The most heat-resistant strain was 17SBSC19 (c-*cpe*), which exhibited a *δ*_95 °C_ value of 47 min, along with the highest sporulation ability (7.9 ± 0.43 log_10_ CFU/mL). These traits make this strain a significant hazard due to its ability to withstand cooking. On the other hand, strains carrying *cpe* on plasmid exhibit a low heat resistance with an average of 2.4 ± 2.44 min at 89 °C [9], 1.77 ± 0.73 min at 100 °C [27] and 12 ± 13 min at 85 °C (our study, Table 4). This heat-sensitive phenotype may explain the low prevalence of these strains (p-*cpe*) in food poisoning cases associated with *C. perfringens* toxinotype F.

The main finding in this study is the identification of a third group with intermediate heat resistance values, which lies between the highly heat-resistant strains (c-*cpe*) and the heat-sensitive strains (p-*cpe*). Strains exhibiting intermediate heat-resistance phenotype are *cpe*-negative and phylogenetically located within the “chromosomal *cpe* clade”. As shown previously, these strains are genetically very close to strains that carry *cpe* chromosomally [7,37]. To our knowledge, our study is the first one to provide heat resistance values for this group of strains, with an average of 69 ± 26 min at 85 °C (Table 4). Indeed, the reviewed literature suggests that *cpe*-negative strains are given less importance since they generally exhibit a heat-sensitive phenotype with *D*_89 °C_-value of 1.5 ± 0.63 min [9] (Table 4) and *D*_95 °C_-value of 2.18 ± 0.46 min [26].

This article highlights the existence of an intermediate heat-resistant group of *cpe*-negative strains and challenges the traditional binary classification (heat-resistant vs. heat-sensitive). It also suggests a new layer of variability in heat stress adaptation. This observation is corroborated by Orsburn et al. (2008) [16], who noted that the distinction between *cpe*-positive and *cpe*-negative strains was not always marked in terms of heat resistance, but without being able to provide an explanation. Other *Clostridium* species are also known to exhibit variable thermal resistance. In *C. botulinum*, heat resistance varies both between and within groups: proteolytic Group I strains produce the most heat-resistant spores (*D*_121.1 °C_ = 0.21 min), whereas non-proteolytic Group II strains are the most heat-sensitive (*D*_80 °C_ = 0.6–1.25 min). Group III strains display *D*_104 °C_ values ranging from 0.1 to 0.9 min, while Group IV strains exhibit *D*_104 °C_ values between 0.8 and 1.12 min [37].

Although *cpe*-negative Type A strains are not generally associated with food poisoning, they possess other virulence factors [1]. For example, *nagH* gene has been identified in *cpe*-negative strains associated with food poisoning and may contribute to their enterotoxigenic potential [1]. This gene encodes a hyaluronidase that increases tissue permeability and facilitates the spread of alpha-toxins [38]. Furthermore, the possibility of their conversion into Type F strains through *cpe* plasmid transfer cannot be ruled out [6]. Pangenome analysis revealed a high degree of variability and genomic plasticity in *C. perfringens*, reflecting frequent events of gene gain and loss [7,39]. Their heat resistance, along with the potential for *cpe* gene transfer, could make these strains a relevant hazard for cooked, cooled, and re-heated meat products.

## 5. Conclusions

This study provides new insights into the phenotypic and genetic diversity of *Clostridium perfringens* regarding its sporulation ability and heat resistance. The results show that strains belonging to “the chromosomal *cpe* clade”, whether carrying the *cpe* gene or not, exhibit high sporulation ability and produce heat-resistant spores. In contrast, strains within the “plasmidic *cpe* clade” demonstrate low sporulation abilities and are heat-sensitive. A key finding of this work is the identification of a group of *cpe*-negative strains with intermediate heat resistance, a phenotype not previously described. This challenges the traditional classification, which strictly opposes heat-resistant c-*cpe* strains and heat-sensitive p-*cpe* strains. It also suggests the existence of unknown mechanisms of stress adaptation that are potentially associated with the genomic and molecular environment of the “chromosomal *cpe* clade” (mostly *cpe*-positive, with some *cpe*-negative), and that are independent of the *cpe* gene.

## Figures and Tables

**Table 2:** Bacterial strains of *C. perfringens* used in this study.

Clade	Strains	Origin	Toxinotype	*cpe*
Chromosomal *cpe* clade ^(a)^	17SBSC19	FBOs, France, 2017, sweet potato and shallot compote, plant-based	F	[+]
	16SBCL940	FBOs, France, 2015, Russian-style chicken, poultry	F	[+]
	NCTC 8239	United Kingdom, 1952, salted beef, bovine	F	[+]
	16SBCL107	FBOs, France, 2013, sautéed turkey, poultry	A	[−]
	16SBCL609	FBOs, France, 2015, vegetables, plant-based	A	[−]
Plasmidic *cpe* clade ^(b)^	16SBCL525	FBOs, France, 2016, vegetables, plant-based	F	[+]
	17SBCL79	FBOs, France, 2017, veal stew in white sauce, bovine	F	[+]
	17SBCL67	FBOs, France, 2017, roast chicken seasoning, plant-based	A	[−]
	16SBCL1078	FBOs, France, 2016, chicken coriander stir-fry, poultry	A	[−]

^(a)^ The “chromosomal *cpe* clade” is a clade that contains mainly *cpe*-positive strains carrying the *cpe* gene on a chromosome (c-*cpe*) and few strains in which the *cpe* gene is absent [1]. ^(b)^ The “plasmidic *cpe* clade” is a clade that contains mainly *cpe*-negative strains and few *cpe*-positive strains with plasmid-mediated *cpe* (p-*cpe*) [1]. [+]: presence of *cpe* gene. [−]: absence of *cpe* gene.

**Table 3:** Estimation of heat resistance parameters (*δ* and *p*-value) after a heat treatment at 85 °C and 95 °C for the control strains NCTC 8239 (ATCC 12917/CIP 104880) and six FBO strains. The mean values of heat resistance parameters for each strain, standard deviation, and goodness of fit indicators were calculated and evaluated using MATLAB R2024b.

Clade	Strains	Origin	Toxinotype	*cpe*	δ_85 °C_ (min) ^(c)^	δ_95 °C_ (min) ^(c)^
Chromosomal *cpe* clade ^(a)^	17SBSC19	FBOs, France, 2017, sweet potato and shallot compote, plant-based	F	[+]	328 ± 136	47 ± 24
	16SBCL940	FBOs, France, 2015, Russian-style chicken, poultry	F	[+]	148 ± 100	55 ± 38
	NCTC 8239	United Kingdom, 1952, salted beef, bovine	F	[+]	155 ± 120	46 ± 14
	Average				210 ± 137	49 ± 20
	16SBCL107	FBOs, France, 2013, sautéed turkey, poultry	A	[−]	62 ± 37	9 ± 4
	16SBCL609	FBOs, France, 2015, vegetables, plant-based	A	[−]	75 ± 15	10 ± 3
	Average				69 ± 26	9.5 ± 3.4
Plasmidic *cpe* clade ^(b)^	16SBCL525	FBOs, France, 2016, vegetables, plant-based	F	[+]	19 ± 15	*
	17SBCL79	FBOs, France, 2017, veal stew in white sauce, bovine	F	[+]	5 ± 8	*
	Average				12 ± 13	*
Numbers of data			34 survival curves			
*p*			0.68 ± 0.06			
*R^2^*			0.99			
*MSE*			0.02			
*RMSE*			0.15			
*AICc*			−105.29			

^(a)^ The “chromosomal *cpe* clade” is a clade that contains mainly *cpe*-positive strains carrying the *cpe* gene on a chromosome (c-*cpe*) and few strains in which the *cpe* gene is absent [1]. ^(b)^ The “plasmidic *cpe* clade” is a clade that contains mainly *cpe*-negative strains and few *cpe*-positive strains with plasmid-mediated *cpe* (p-*cpe*) [1]. ^(c)^ Data were obtained from three independent spores batches produced in the same standard conditions for each strain. [+]: presence of *cpe* gene. [−]: absence of *cpe* gene. *: Not determined.

**Table 4:** Review of heat resistance value obtained for different *C. perfringens* strains by different authors. The results obtained in this study have been included in the table.

Strain	TOXINOTYPE	*cpe*	*cpe* Location	Origin	Year of Isolation	Region/Country	D_100 °C_ (min) ^(a)^	δ_95 °C_ (min) ^(b)^	D_89 °C_ (min) ^(a)^	δ_85 °C_ (min) ^(b)^	Average and Stand Deviation [Study]
NCTC8239	F	(+)	Chromosome	Food (salted beef) Food poisoning	1952	The United Kingdom	43.3				D_100 °C_ (min) ^(a)^ = 53.3 ± 10.03 [27]
191–10	F	(+)	Chromosome	Human	1990	The United States	64.3				
FD1041	F	(+)	Chromosome	Human	1980	The United States	49.7				
E13	F	(+)	Chromosome	Human	1960	The United States	45.5				
NCTC10239	F	(+)	Chromosome	Human	1950	Europe	63.7				
17SBSC19	F	(+)	Chromosome	Plant-based, food poisoning	2017	France		47			δ_95 °C_ (min) ^(b)^ = 49 ± 20 [This study]
16SBCL940	F	(+)	Chromosome	Poultry, food poisoning	2015	France		55			
NCTC8239	F	(+)	Chromosome	Food (salted beef), Food poisoning	1952	The United Kingdom		46			
AAD1900a	F	(+)	Plasmide	Feces, human, antibiotic associated diarrhea	2002	Finland			3.07		D_89 °C_ (min) ^(a)^ = 2.4 ± 2.44 [9]
CPI 18-1b	F	(+)	Plasmide	Feces, human, healthy	2003	Finland			0.56		
CPI 39-1a	F	(+)	Plasmide	Feces, human, healthy	2003	Finland			1.55		
CPLi 6-1	F	(+)	Plasmide	Sludge	2007	Finland			1.53		
CPM 77b	F	(+)	Plasmide	Soil	2000	Finland			0.59		
C216	F	(+)	Plasmide	Roast beef, Food poisoning	2006	Finland			0.92		
149/92	F	(+)	Plasmide	Feces, Food poisoning	1992	Germany			6.42		
AAD 1527a	F	(+)	Plasmide	Feces, human, antibiotic associated diarrhea	2002	Finland			5.10		
AAD1903	F	(+)	Plasmide	Feces, human, antibiotic associated diarrhea	2002	Finland			1.03		
CPI 53k-r1	F	(+)	Plasmide	Feces, human, healthy	2003	Finland			0.50		
CPI 63K-r5	F	(+)	Plasmide	Feces, human, healthy	2003	Finland			1.28		
CPI 75-1	F	(+)	Plasmide	Feces, human, healthy	2003	Finland			8.05		
CPLi3-1	F	(+)	Plasmide	Sludge	2003	Finland			0.38		
721/84	F	(+)	Plasmide	Rabbit meat	1984	Germany			3.10		
16SBCL525	F	(+)	Plasmide	Plant-based, food poisoning	2016	France				19	δ_85 °C_ (min) ^(b)^ = 12 ± 13 [This study]
17SBCL79	F	(+)	Plasmide	Bovine, food poisoning	2017	France				5	
CPI 18-6	A	(−)	*	Feces, human, healthy	2003	Finland			0.84		D_89 °C_ (min) ^(a)^ = 1.5 ± 0.63 [9]
CPN 17a	A	(−)	*	Cattle feces	2000	Finland			2.08		
CPS 2a	A	(−)	*	Pig feces	2000	Finland			1.65		
16SBCL107	A	(−)	*	Poultry, food poisoning	2013	France				62	δ_85 °C_ (min) ^(b)^ = 69 ± 26 [This study]
16SBCL609	A	(−)	*	Plant-based, food poisoning	2015	France				75	

^(a)^ Log-linear model was used for heat resistance estimation. ^(b)^ Model based on Weibul distribution was used for heat resistance estimation. (+): presence of *cpe* gene. (−): absence of *cpe* gene. *: Not determined.

## Data Availability

The original contributions presented in this study are included in the article. Further inquiries can be directed to the corresponding author.

## Abbreviations

The following abbreviations are used in this manuscript:

FBO	Food-Borne outbreaks
CPE	Clostridium perfringens enterotoxin
c-*cpe*	chromosomal cpe
p-*cpe*	plasmidic cpe
